# Pathogenicity of novel goose-origin astrovirus causing gout in goslings

**DOI:** 10.1186/s12917-020-02739-z

**Published:** 2021-01-20

**Authors:** Dan Yin, Jiajun Tian, Jing Yang, Yi Tang, Youxiang Diao

**Affiliations:** 1 College of Animal Science and Technology, Shandong Agricultural University, 61 Daizong Street, Tai’an, 271018 Shandong China; 2 Shandong Provincial Key Laboratory of Animal Biotechnology and Disease Control and Prevention, Tai’an, 271018 Shandong China; 3 Shandong Provincial Engineering Technology Research Center of Animal Disease Control and Prevention, Tai’an, 271018 Shandong China

**Keywords:** Novel goose-origin astrovirus, Goslings, Gout, Histopathology, Biochemical parameters, Viral loads

## Abstract

**Background:**

A novel goose-origin astrovirus (GoAstV) has broken out across China in recent years, causing gout in goslings with a mortality rate of around 50%. However, our understanding of the dynamic distribution, tissue tropism and pathogenesis of GoAstV is incomplete. In order to assess its pathogenicity, one-day-old goslings were inoculated separately with GoAstV via oral and subcutaneous injection routes.

**Results:**

Clinical symptoms, gross and microscopic lesions, blood biochemical parameters and viral loads were detected and recorded for 20 days after infection. Typical gout was observed in experimental goslings. GoAstV can be replicated in tissues and cause pathological damage, especially in the kidney, liver, heart and spleen. Virus-specific genomic RNA was detected in blood, cloacal swabs and all representative tissues, and virus shedding was detected up to 20 days after inoculation, suggesting that GoAstV has a wide tissue tropism and spread systematically after inoculation. The viral copy numbers examined in kidney were the highest, followed by spleen and liver.

**Conclusion:**

This experiment determined the accurate value of viral loads and biochemical indicators of GoAstV-induced goslings. These findings increase our understanding of the pathogenicity of GoAstV in goslings and provide more reference for future research.

## Background

Astroviruses are non-enveloped positive-sense single-stranded RNA viruses belonging to the *Astroviridae* family [1]. Currently, the family was classified into two genera, *Mamastroviruses* and *Avastroviruses* [2]. Astroviruses are characterized with a size of approximately 30 nm in diameter [3]. The length of genomes ranges from 6.1 to 7.9 kb, consisting of a 5′-untranslated region (UTR), three open reading frames (ORFs), 3′-UTR and a poly (A) tail [4]. ORF 1a and ORF 1b encode non-structural protein for genomic replication, and ORF 2 encodes capsid proteins associated with the virion formation [5]. The astrovirus polymerase has no proofreading function during the replication process, so the replication of the virus is prone to error, resulting in an increase in the mutation rate of the virus [6]. The high genetic diversity and recombination potential of astroviruses indicate that cross-species transmission may be frequent [7, 8].

Since the initial discovery in faeces of infants in 1975, astroviruses have been detected in many species, including poultry [9–12]. Infection with astroviruses mainly causes acute gastroenteritis in human and animals, occasionally with encephalitis [8, 13]. In poultry, astroviruses infection has been found to be associated with a variety of diseases, such as nephritis in chicken, visceral gout in broilers and fatal hepatitis in ducklings, leading to severe economic losses [14–16]. The typical characteristics of astroviruses infection in poultry are high morbidity and mortality, low feed conversion rate and growth retardation [4].

Since November 2016, a new disease in goslings, of which the specific symptoms are gout, hemorrhages and renomegaly, has spread to many provinces of China, including Shandong, Jiangsu and Anhui, as well as other regions. It is estimated that the economic losses caused by the epidemic are between 1.2–1.5 billion yuan [17]. The infection rate of the disease was usually up to 80%, and the mortality rate even up to 50% [18]. The infection can be identified from the goslings aged from 5 to 20 days. Autopsies showed that severe urate deposition in the internal organs, especially the heart, liver and kidney. A novel goose-origin astrovirus (GoAstV) has been identified in infected goslings, which is different from previously reported avian astroviruses in terms of genotypes and has a nucleotide homology of less than 70% of other avian astroviruses [17, 19–21]. In the present study, we detect the dynamic distribution of GoAstV in goslings infected by oral and subcutaneous injection routes respectively, which revealed virus shedding and virus distribution, helping to further understand the pathogenicity and transmission mechanism. It provided theoretical support for the prevention and control of the disease.

## Results

### Clinical signs, body weight and gross lesions

**Fig. 1:**
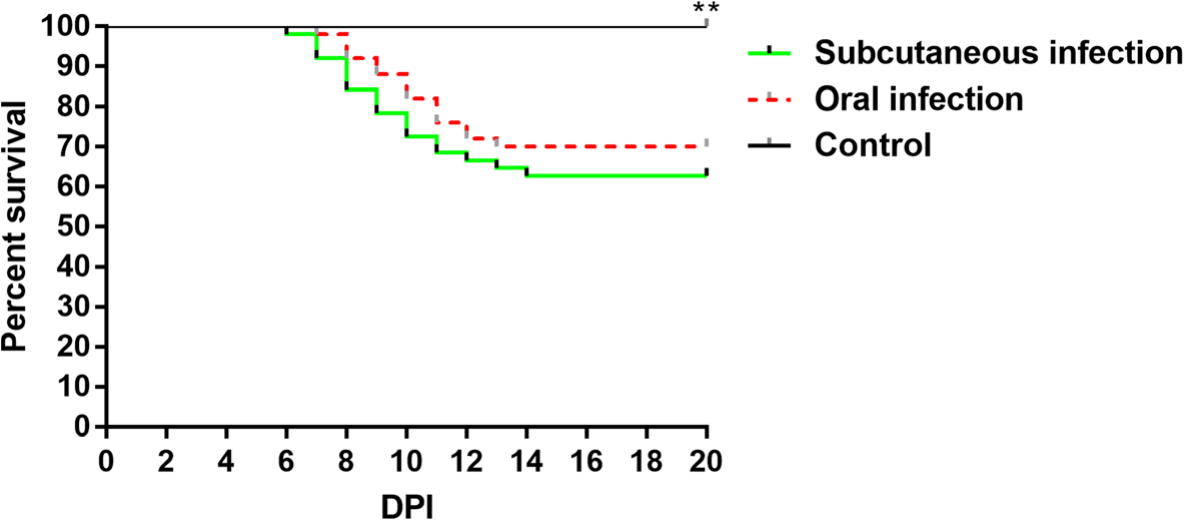
Survival rates of goslings after inoculation with SDPY strain. The percentage of goslings that survived in the infected groups were extremely significantly lower than in the control group. **p < 0.01. **Means that the difference between infection groups and control group was extremely significant

**Fig. 2:**
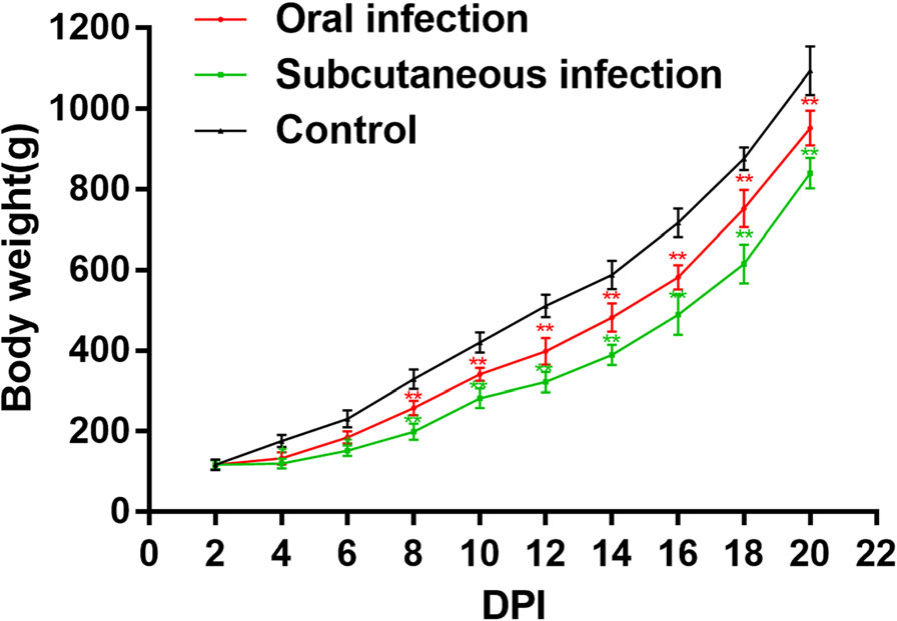
Weight changes of goslings after infection with GoAstV-SDPY strain. The error bars indicate means ± standard deviation (SD). The comparison was between infection groups and control group. *p < 0.05 and **p < 0.01. The same as below. *Means that the difference between infection groups and control group was significant. **Means that the difference between infection groups and control group was extremely significant

The goslings in oral infection group began to present loss of appetite and depression at 5 dpi. Compared with oral infection group, those clinical signs were observed as early as 4 dpi in subcutaneous infection group. Survival curves of goslings were performed using Prism 7 program (GraphPad) (Fig. 1). Goslings in oral infection group began to die at 7 dpi and presented a mortality 30% at 13 dpi. While goslings in subcutaneous infection group began to die at 6 dpi and exhibited a mortality of 38% at 14 dpi. The survival rate showed extremely significant difference between the infected groups and the control group at the end of observation period (*P* < 0.01). Compared with the control group, goslings in the infected groups grew slowly and gained less weight. In addition, the body weights of goslings in the subcutaneous infection group were lower than those in the oral infection group (Fig. 2).

**Fig. 3:**
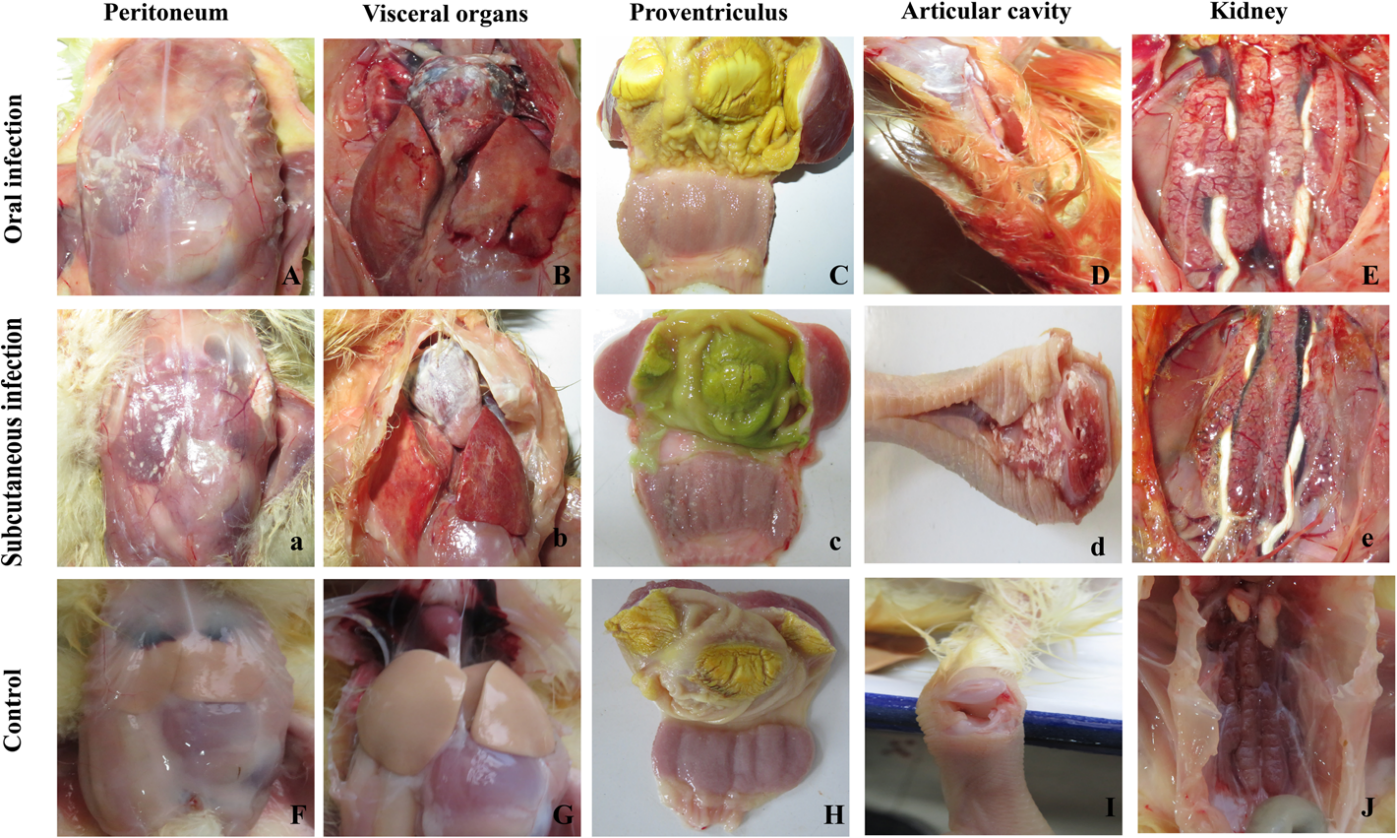
Postmortem lesions of goslings that died at 7 dpi. Deposition of urate in the peritoneum (A, a), visceral organs (B, b), proventriculus (C,c) and articular cavity (D, d). Severe haemorrhage and swellings of kidneys and urate deposits in ureters (E, e). F, G, H, I and J are non-infected control

The lesions of all dead goslings in experimental groups had a high similarity. At necropsy, gout with urate deposits in visceral organs and articular cavity, severe hemorrhages and renal enlargement, and white substance-filled ureters can be observed in dead goslings (Fig. 3). No mortality or any clinical symptoms were found in control goslings in this experiment.

### Histopathology in different tissues

**Fig. 4:**
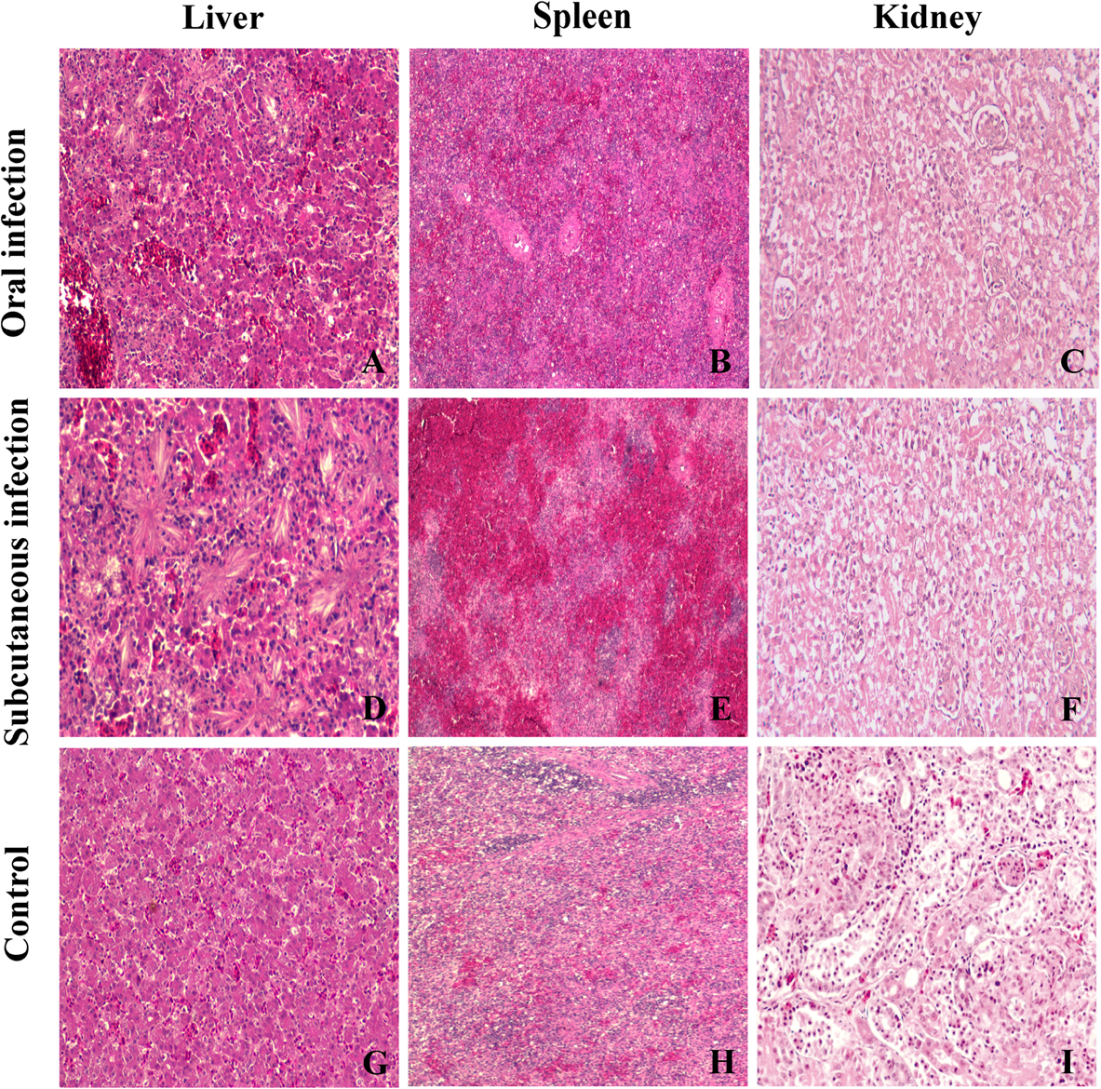
Histopathology lesions of tissues from goslings infected with GoAstV at 7 dpi. HE-stained liver section showed urate crystals and necrosis (A, D). HE-stained spleen section showed diffuse haemorrhage (B, E). HE-stained kidney section, renal tubular epithelial cell degeneration, necrosis and exfoliation (C, F). G H and I are negative control (histology changes in healthy goslings). Magnification, × 100

Histopathological analysis showed that there were obvious and almost identical histopathological changes in both experimental groups at 7 dpi. And the most obvious lesions of this disease were in liver, spleen and kidney (Fig. 4). Colorless to basophilic radiating, sharp, acicular, crystalline deposits (urate tophi) are often surrounded by low to moderate numbers of viable and degenerate heterophils, small amounts of eosinophilic cellular and karyorrhectic debris (necrosis), which were frequently observed in the livers (Fig. 4a, d). The spleen showed diffuse hemorrhage (Fig. 4b, e). As showed in Fig. 4c and f, renal tubular epithelial cell degeneration, necrosis and exfoliation were prominent features in infected goslings in our experiment. In summary, no lesions were observed in the corresponding tissues of goslings in the control group, while similar lesions were observed in various tissues of goslings in both infected groups.

### Detection of biochemical parameters

**Fig. 5:**
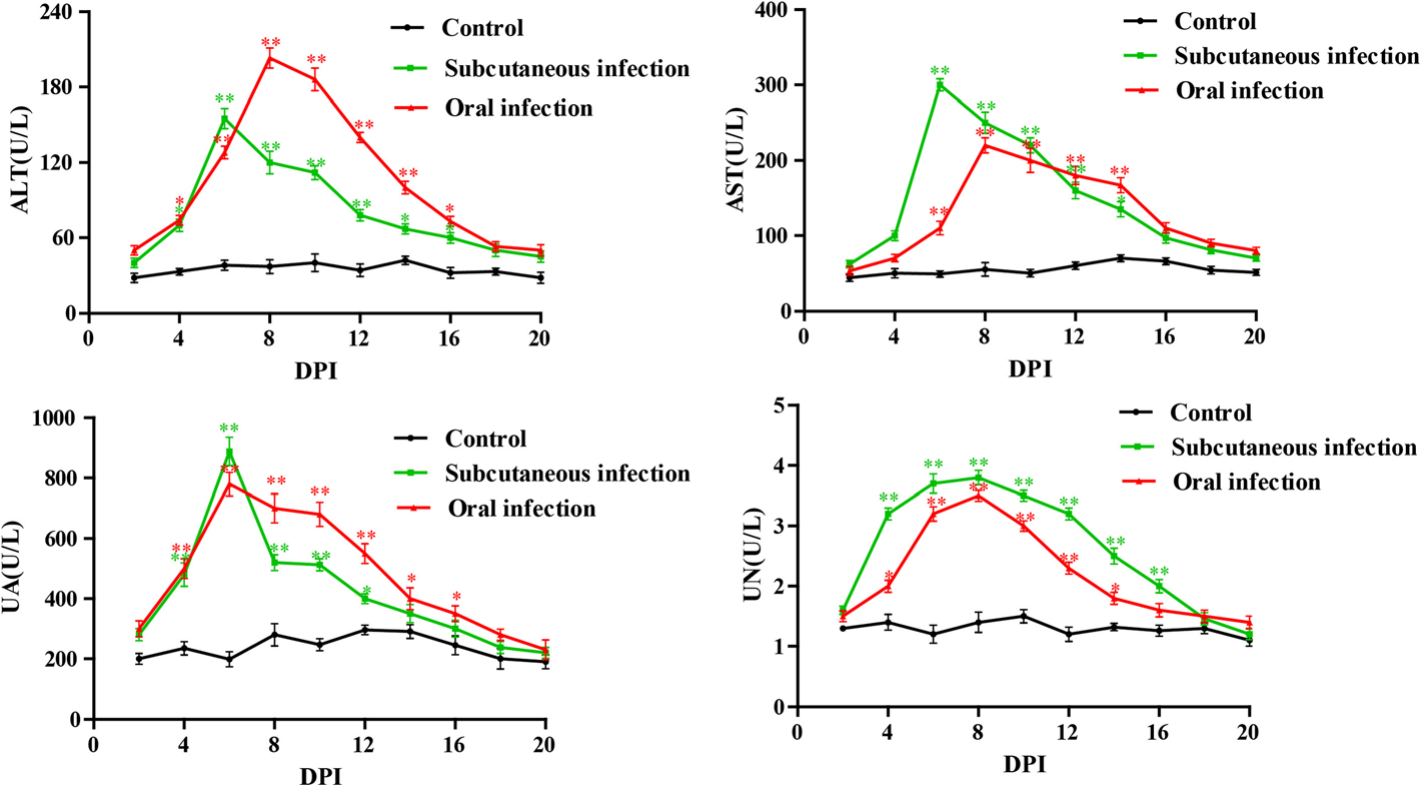
Dynamics of ALT, AST, UA and UN in serum of the goslings post artificially infected with GoAstV. *Means that the difference between infection groups and control group was significant. **Means that the difference between infection groups and control group was extremely significant

The detection results of three groups were presented in Fig. 5. The analysis suggested that the ALT, AST, UA and UN of infected groups were higher than that of control group. Briefly, ALT, AST and UA of subcutaneous infection group all reached the peak at 6 dpi, UN at 8 dpi. ALT, AST and UN of oral infection group all reached the peak at 8 dpi, UA at 6 dpi. After reaching peak levels, the above biochemical parameters declined with time.

### Detection of viral load in the blood

**Fig. 6:**
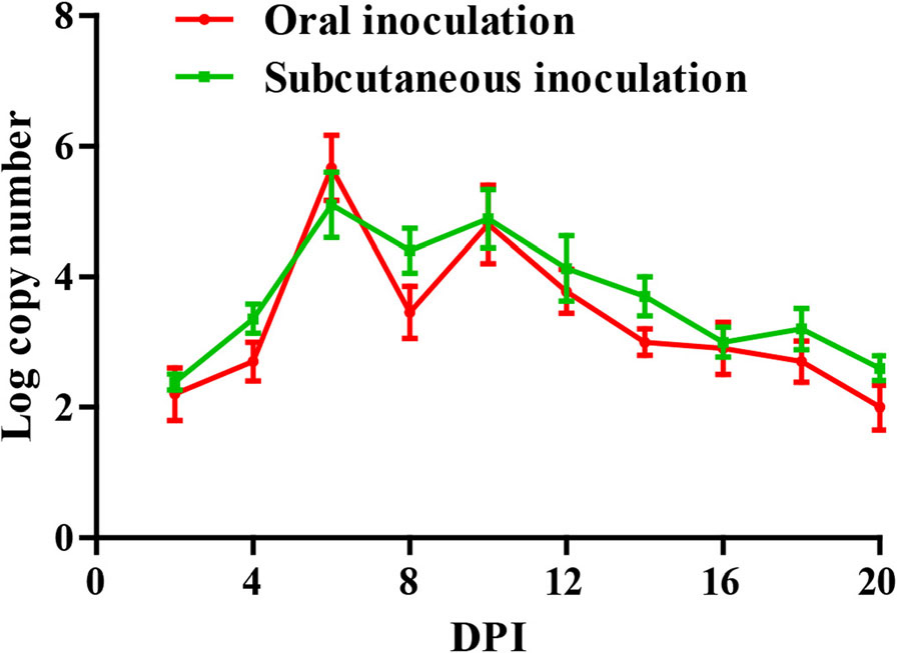
The viral copy numbers in blood of goslings infected with GoAstV. Double replication peaks occurred in the blood at 6 dpi and 10 dpi

The detection results of GoAstV copy number in blood were shown in Fig. 6. Note that the viral RNA was not detected in the control group. Viral copy numbers of both infected groups could be detected in the blood as early as 2 dpi and reached double replication peaks at 6 dpi and 10 dpi. Subsequently, viral load declined with time. The changes of viral load in blood were almost the same in both infected groups.

### Detection of viral load in cloacal swabs

**Fig. 7:**
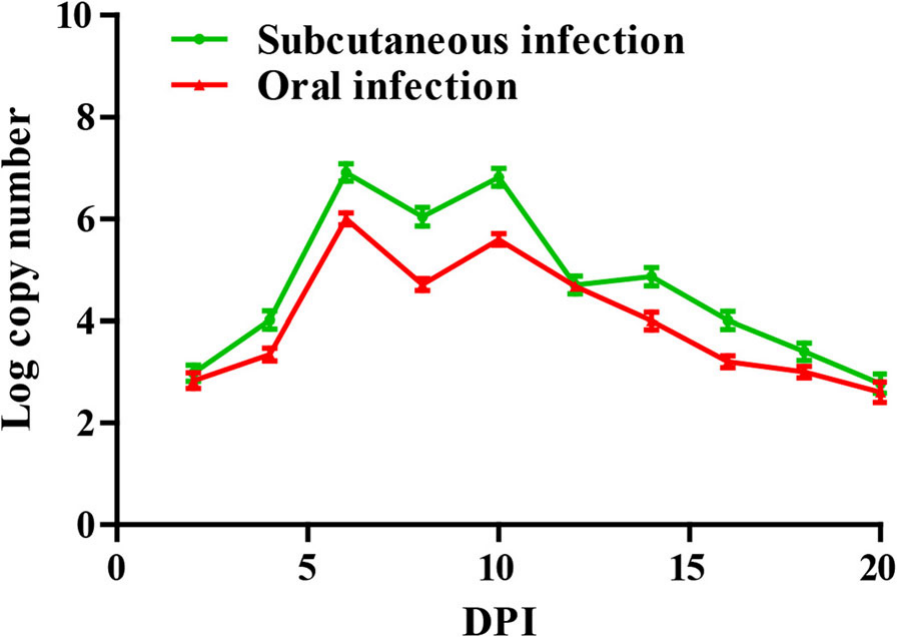
The viral copy numbers in cloacal swabs of goslings infected with GoAstV. Double replication peaks occurred in the cloacal swabs at 6 dpi and 10 dpi

The detection results of GoAstV copy number in cloacal swabs were shown in Fig. 7. Viral copy numbers of both infected groups reached double replication peaks at 6 dpi and 10 dpi. Moreover, viral copy numbers of subcutaneous infection group were higher than that of oral infection group.

### Detection of viral load in tissues

**Fig. 8:**
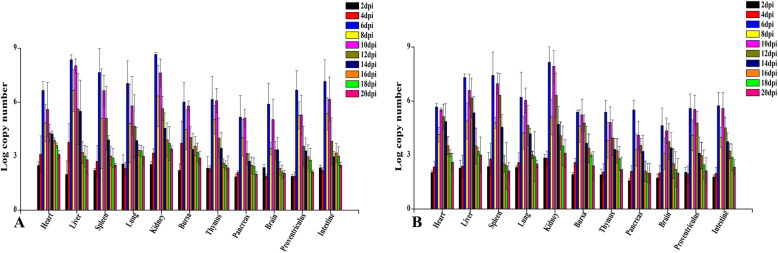
Dynamic distribution of GoAstV in experimentally infected goslings. (A) The viral copy numbers in tissue samples of subcutaneous infection group. (B) The viral copies in tissue samples of oral infection group. Double replication peaks occurred in all investigated tissues at 6 and 10 dpi. More viral copy numbers were found in the kidneys than in other tissues

The dynamic changes of GoAstV in different tissues of goslings were detected by qRT-PCR (Fig. 8). The virus RNA can be detected throughout the experiment and attained high levels in all investigated tissues at 6 and 10 dpi. Maximum virus copy numbers were achieved at 6 dpi. Notably, more viral copy numbers were found in the kidneys than in other tissues. Furthermore, virus copy numbers in all investigated tissues showed similar trend in both infected groups. No positive viral RNA was recorded in the control goslings in this study.

## Discussion

In recent years, increasing clinical cases of GoAstV infections have been concerned and are reported in different provinces of China, inducing huge economic losses for goose industry [18, 19]. Although many studies have been conducted on virus isolation and characterization of GoAstV, less is known about the pathogenicity and the replicator dynamics in vivo, which is critical for enclosing the pathogenesis of the emerged GoAstV. So, in this study, a systematic trial of the pathogenicity of GoAstV in goslings was carried out.

As expected, this study revealed that goslings infected with GoAstV showed clear symptoms of gout. Goslings in subcutaneous infection group could have been symptomatic much more or earlier than those in oral infection group. Severe and identical lesions were observed in both infection groups, suggesting that GoAstV could infect goslings horizontally through the digestive tract. The results laid the foundation for us to better understand the route of GoAstV transmission. GoAstV-infected goslings had severe urate deposits on the internal organs and ureters. These histopathological observations of GoAstV-induced renal tubular epithelial cell degeneration and necrosis in consistency with the previous study that the Moser reported in 2007, which provided further evidence for the impaired renal function [22]. In experimental groups, severe urate deposits may have blocked the renal tubules and caused renal damage [23], thereby reducing uric acid excretion and increasing serum uric acid. Uric acid accumulates in the blood and can be transferred to any of the organs of the body through the blood circulation. Therefore, organs with larger blood flow, such as heart, liver and kidney, have more obvious gout lesions. Furthermore, the clinical symptoms and histological lesions in goslings were largely consistent with the other latest reports [1, 19]. These results indicated that GoAstV could cause kidney damage, which maybe the main cause of gosling gout in China. Apart from gout, GoAstV infection can decrease feed intake and lower down feed conversion efficiency, which leads to growth repression. The weight loss of infected goslings is an important problem, because they lost 37% of their weight at 14 dpi, which caused considerable economic losses.

AST mainly exists in cardiomyocytes, followed by hepatocytes [24]. ALT is present in high concentration in hepatocytes, it’s the specific marker of hepatocellular injury because it occurs exclusively in the liver [25]. If hepatic cells or myocardial cells are damaged, above enzymes will leak into the blood and levels of these will increase rapidly. It is reported that markedly elevated serum UA is clearly associated with gouty arthritis and nephrolithiasis [26]. UA and UN levels can reflect tissue damages, especially for renal impairment [27]. The detection results of this study showed that almost all blood biochemical parameters of experimental groups were higher than those of control group and then decreased with time. One such factor may be tissue and cells damage caused by the invasion of virus, resulting in the leakage of enzymes into the circulation. And we know that tissue damage is able to self-repair. Therefore, the subsequent reduction in the level of enzyme is likely caused by the cellular self-repairing capability. This also supports changes in the above histopathological damage.

Related to the qRT-PCR data, viral loads could be detected in the blood, cloacal swabs and various organs collected from infected goslings at 2 dpi, indicating the primary viremia can be developed less than 48 h post infection. Viral RNA was found in all investigated tissues sampled from the infected goslings suggesting that GoAstV has a wide tissue tropism and spread systematically after inoculation, which was in accord with those in other studies [1]. GoAstV was continuously detected in infected goslings throughout the experiment indicating that the virus can replicate efficiently in vivo. The kidney may be one of the main target organs of GoAstV due to the highest virus copy number, which further indicated that GoAstV can cause gout owning to the impaired kidney. In addition, target organs may also include spleen and liver, and they could be used as options for detection of GoAstV due to high viral copy numbers. In this study, the changes of viral loads in cloacal swabs, blood and various organs were basically the same. Reasonable immunization procedures can be formulated according to the shedding virus in production practice. During the peak period of viral loads, the disinfection of goose house should be strengthened to prevent the wide spread of the disease.

## Conclusions

The commercial vaccines against GoAstV have not yet been developed in China due to a lack of knowledge of the relevant diseases. Therefore, further pathogenic analysis of GoAstV is necessary and would provide useful information for the development of the efficient vaccine against gout. In conclusion, this experiment determined the accurate value of viral loads and biochemical indicators of GoAstV-induced goslings. It fills in the gaps in this aspect of data and provides more reference for future research.

## Methods

### Animals

One hundred fifthy one-day-old healthy goslings were purchased from a commercial hatchery in Shandong Province. Three goslings from each group were randomly selected every 2 days after inoculation for the test. We only needed 30 goslings for each group, and in the actual trial we prepared 50 goslings much more than we planned. Those goslings were fed ad libitum in specific pathogen-free (SPF) isolators without any immunization. Cloacal and tracheal swabs were collected from goslings before inoculation to ensure that goslings were GoAstV-negative by TaqMan-based one-step real-time quantitative reverse transcriptase-polymerase chain reaction (qRT-PCR) assay.

### Virus

The SDPY strain (accession number: MH052598) used as the challenge virus in this study was isolated in our laboratory from the kidney of goslings with gout [17, 18]. The titer of challenge virus was determined as 10^5.25^ median embryo lethal dose (ELD_50_)/0.2 mL by infection of goose embryos and calculation of the titer by the Reed and Muench method [28].

### Pathogenicity assessment of GoAstV in goslings

To determine the pathogenicity of GoAstV, 150 one-day-old goslings were randomly divided into three groups of 50 and housed in separate SPF isolators. The goslings in the experimental groups were inoculated with 0.2 mL of GoAstV (SDPY strain; 10^5.25^ ELD_50_/0.2 mL) either orally or by subcutaneous injection in the neck. The control group was inoculated with equal doses of phosphate buffer saline (PBS) at the same injection site. Water and food were autoclaved before feeding and automatically refilled. Clinical symptoms, gross and microscopic lesions were recorded after infection. On the 2nd, 4th, 6th, 8th, 10th, 12th, 14th, 16th, 18th, and 20th days post-inoculation (dpi), three goslings in each group were randomly selected for weighing. At the same time, serum samples were collected from all selected goslings and stored at − 20 °C, which were used to detect and record the changes of biochemical parameters including alanine aminotransferase (ALT)、aspartate aminotransferase (AST)、urea nitrogen (UN) and uric acid (UA). The biochemical parameters were detected by Taian City Central Hospital (Taian, China). Blood specimens, cloacal swabs and tissue samples of heart, liver, spleen, lung, kidney, bursa, thymus, pancreas, brain, proventriculus and intestine were also collected at each dpi and stored at − 80 °C until were using for detecting and recording the changes of viral load by qRT-PCR. At the end of this study, the remaining geese were euthanized by intravenous injection of sodium pentobarbital (100 mg/kg body weight). The preferred method of euthanasia is intravenous administration of excessive barbiturates, which can suppress the central nervous reflex and render the animal unconscious. Excessive doses of barbiturates can cause the animal to stop breathing, followed by cardiac arrest.

### Histopathology

The goslings that died after inoculation were autopsied and tissue samples (liver, spleen and kidney) were collected for histopathological analysis. Tissue samples were fixed in 10% formalin for 48 h at room temperature, and then routinely processed, embedded in paraffin wax, and cut into 5-μm sections. The sections were stained with hematoxylin and eosin (HE) and examined using light microscopy.

### RNA extraction and qRT-PCR

RNA was extracted by MiniBEST Universal RNA Extraction Kit (TaKaRa, Dalian, China) following the manufacturer’s instructions. The concentration of each RNA sample was measured using the DeNovix DS-11 Spectrophotometer. To determine the viral load in each sample, GoAstV was detected by qRT-PCR method previously established in our laboratory. A pair of specific primers and probe used in this study are as follows, forward: GGTGGGCTAATAACGGAACTCAG, reverse: GACCTATTTCCTTGCGGATCAC and probe: TCGGCTCAACATCGCTGATGGG. The qRT-PCR assay was developed and validated using the LightCycler (Roche Diagnostics) and TaKaRa One Step PrimeScript™ RT-PCR Kit (TaKaRa, Dalian, China). The optimized qRT-PCR reaction volume was 20 μL containing 10 μL 2 × One Step RT-PCR Buffer III, 0.4 μL TaKaRa Ex Taq HS, 0.4 μL PrimeScript RT Enzyme MixII, 0.4 μL forward primer, 0.4 μL reverse primer, 0.4 μL probe, 6.0 μL RNase Free ddH_2_O, and 2.0 μL RNA template. An initial reverse transcription at 45 °C for 5 min, reverse transcriptase inactivation at 95 °C for 10 s, followed by 45 cycles at 95 °C for 5 s and at 60 °C for 20 s, and ending at 4 °C. Fluorescence signals for each sample were harvested at the end of each step at 60 °C.

### Statistical analysis

Each experiment was repeated three times independently and average values were taken. No data exclusion in the manuscript. All data were presented at means ± standard deviation and analyzed using the one-way analysis of variance (ANOVA) procedure of GraphPad Prism 7.0 (GraphPad Software Inc., San Diego, CA, United States). Statistical significance was set at *P* < 0.05.

## Data Availability

The datasets used and/or analyzed in the current study are available from the corresponding author on reasonable request.

## Abbreviations

GoAstV: Goose-origin astrovirus
SPF: Specific pathogen-free
qRT-PCR: Real-time quantitative reverse transcriptase-polymerase chain reaction
ELD_50_: Median embryo lethal dose
PBS: Phosphate buffer saline
dpi: Days post-inoculation
ALT: Alanine aminotransferase
AST: Aspartate aminotransferase
UN: Urea nitrogen
UA: Uric acid
HE: Hematoxylin and eosin
ANOVA: Analysis of variance

## References

[1] Zhang Q, Cao Y, Wang J, Fu G, Sun M, Zhang L, Meng L, Cui G, Huang Y, Hu X (2018). Isolation and characterization of an astrovirus causing fatal visceral gout in domestic goslings. Emerg Microbes Infect.

[2] Qin Y, Fang Q, Liu H, Ji C, Chen Y, Ouyang K, Wei Z, Huang W (2018). Construction of a reverse genetic system for porcine astrovirus. Arch Virol.

[3] Madeley CR (1979). Comparison of the features of astroviruses and caliciviruses seen in samples of feces by electron microscopy. J Infect Dis.

[4] Pantin-Jackwood MJ, Strother KO, Mundt E, Zsak L, Day JM, Spackman E (2011). Molecular characterization of avian astroviruses. Arch Virol.

[5] Arias CF, DuBois RM. The Astrovirus Capsid: A Review. Viruses. 2017:**9**(1). 10.3390/v9010015 PMC5294984 28106836

[6] Tang Y, Murgia MV, Ward L, Saif YM (2006). Pathogenicity of Turkey astroviruses in Turkey embryos and poults. Avian Dis.

[7] Donato C, Vijaykrishna D. The Broad Host Range and Genetic Diversity of Mammalian and Avian Astroviruses. Viruses. 2017:**9**(5). 10.3390/v9050102 PMC5454415 28489047

[8] Mendenhall IH, Smith GJ, Vijaykrishna D (2015). Ecological drivers of virus evolution: Astrovirus as a case study. J Virol.

[9] Appleton H, Higgins PG (1975). Letter: viruses and gastroenteritis in infants. Lancet.

[10] De Benedictis P, Schultz-Cherry S, Burnham A, Cattoli G (2011). Astrovirus infections in humans and animals - molecular biology, genetic diversity, and interspecies transmissions. Infect Genet Evol.

[11] Madeley CR, Cosgrove BP (1975). Letter: 28 nm particles in faeces in infantile gastroenteritis. Lancet.

[12] Sajewicz-Krukowska J, Domanska-Blicharz K (2016). Nearly full-length genome sequence of a novel astrovirus isolated from chickens with 'white chicks' condition. Arch Virol.

[13] Boujon CL, Koch MC, Wuthrich D, Werder S, Jakupovic D, Bruggmann R, Seuberlich T (2017). Indication of cross-species transmission of Astrovirus associated with encephalitis in sheep and cattle. Emerg Infect Dis.

[14] Baxendale W, Mebatsion T (2004). The isolation and characterisation of astroviruses from chickens. Avian Pathol.

[15] Bulbule NR, Mandakhalikar KD, Kapgate SS, Deshmukh VV, Schat KA, Chawak MM (2013). Role of chicken astrovirus as a causative agent of gout in commercial broilers in India. Avian Pathol.

[16] Chen L, Xu Q, Zhang R, Li J, Xie Z, Wang Y, Zhu Y, Jiang S (2012). Complete genome sequence of a duck astrovirus discovered in eastern China. J Virol.

[17] Yang J, Tian J, Tang Y, Diao Y (2018). Isolation and genomic characterization of gosling gout caused by a novel goose astrovirus. Transbound Emerg Dis.

[18] Niu X, Tian J, Yang J, Jiang X, Wang H, Chen H, Yi T, Diao Y (2018). Novel goose Astrovirus associated gout in Gosling, China. Vet Microbiol.

[19] Zhang X, Ren D, Li T, Zhou H, Liu X, Wang X, Lu H, Gao W, Wang Y, Zou X (2018). An emerging novel goose astrovirus associated with gosling gout disease, China. Emerg Microbes Infect.

[20] Yuan X, Meng K, Zhang Y, Yu Z, Ai W, Wang Y (2019). Genome analysis of newly emerging goose-origin nephrotic astrovirus in China reveals it belongs to a novel genetically distinct astrovirus. Infect Genet Evol.

[21] Zhang Y, Wang F, Liu N, Yang L, Zhang D (2017). Complete genome sequence of a novel avastrovirus in goose. Arch Virol.

[22] Moser LA, Carter M, Schultz-Cherry S (2007). Astrovirus increases epithelial barrier permeability independently of viral replication. J Virol.

[23] Mustafa S, Alsughayer A, Elgazzar A, Elassar A, Al Sagheer F (2014). Effect of sulfa drugs on kidney function and renal scintigraphy. Nephrology (Carlton).

[24] Fan S, Zheng J, Duan Z, Yang N, Xu G (2014). The influences of SE infection on layers' production performance, egg quality and blood biochemical indicators. J Anim Sci Biotechnol.

[25] Kew MC (2000). Serum aminotransferase concentration as evidence of hepatocellular damage. Lancet.

[26] Kanbay M, Solak Y, Dogan E, Lanaspa MA, Covic A (2010). Uric acid in hypertension and renal disease: the chicken or the egg?. Blood Purif.

[27] Duru M, Nacar A, Yonden Z, Kuvandik G, Helvaci MR, Koc A, Akaydin Y, Oksuz H, Sogut S (2008). Protective effects of N-acetylcysteine on cyclosporine-A-induced nephrotoxicity. Ren Fail.

[28] Matumoto M (1949). A note on some points of calculation method of LD50 by reed and Muench. Jpn J Exp Med.

